# Decision Support System Applications for Scheduling in Professional Team Sport. The Team's Perspective

**DOI:** 10.3389/fspor.2021.678489

**Published:** 2021-06-04

**Authors:** Xavier Schelling, Jose Fernández, Patrick Ward, Javier Fernández, Sam Robertson

**Affiliations:** ^1^Institute for Health and Sport, Victoria University, Melbourne, VIC, Australia; ^2^Faculty of Health Sciences, School of Behavioral and Health Sciences, Australian Catholic University, Fitzroy, VIC, Australia; ^3^Human Performance Research Centre, Faculty of Health, University of Technology Sydney (UTS), Sydney, NSW, Australia; ^4^Futbol Club Barcelona, Barcelona, Spain; ^5^Department of Computer Science, Universitat Politecnica de Catalunya, Barcelona, Spain

**Keywords:** decision making, information system, sport science, optimization, scheduling

## Abstract

**Background:** Periodization implies the systematic planning of training and competition with the goal of reaching the best possible performance in the most important competition. In team sports, this consists of finding a flight-and-practice schedule that maximizes the opportunities to perform the periodized contents (e.g., trips, practices, games, and days off). This process is conducted whilst considering known constraints (e.g., competitive schedule, roster availability, weather, especial events, holidays, or emotional effect of days away). The way a scheduling decision support system (DSS) leads users to make a decision should allow for flexibility, whilst minimizing users' confusion and facilitating the understanding of the recommendation given by the scheduling decision support system. Traditional approaches to solving scheduling problems use either simulation models, analytical models, heuristic approaches or a combination of these methods. When it comes to evaluate how the scheduling DSS is performing, three overarching aspects need to be reviewed: context satisfaction, process efficiency, and output quality. Appropriate training periodization and scheduling of trips and training sessions are critical for teams to optimize training and recovery processes in order to maximize health and performance. This article presents a methodological framework for designing decision-support systems for scheduling in professional team sports.

## Introduction

Professional sport leagues involve millions of fans, broadcast rights, merchandizing, and advertising. Therefore, they constitute a major economic activity, where revenue maximization and logistical optimization are key factors (Kendall et al., 2010). Consequently, in popular team sports such as soccer, basketball, baseball, or ice hockey, it is common to have several games per week (i.e., ≥3) per team throughout a competitive season. Additionally, any professional sport requires training and traveling periodically, which should be periodized considering the competitive calendar. Periodization implies the systematic planning of training and competition with the goal of reaching the best possible performance in the most important competition of the season (Robertson and Joyce, 2018). This goal involves the development and optimization of the multiple factors that drive sport performance, which rely on psychological and physiological processes (e.g., fitness, cognition, and emotions), as well as environmental conditions (e.g., weather, equipment, rewards) (Seirul·lo, 1998).

Most professional team sports globally utilize a tournament format where each team plays against every other a fixed number of times (also known as “all-play-all” or “round robin tournament”) (Ribeiro, 2012; Byl, 2013). Every team has prior knowledge of opponents along with, the date, location and time in which they will compete, which provides an opportunity to prepare for both the tournament and upcoming games (Byl, 2013). In some leagues (e.g., National Basketball Association—NBA), the exact day and time for all games is released before the season starts, in others (e.g., La Liga) they are defined throughout the season, for instance five games in advance. Each team typically has its own venue at its home city and each game is played at the venue of either one of the two teams in confrontation.

The timing of a national league season (i.e., domestic league) must be coordinated with international competitions such as World Cups, Olympic Games, Eurocup, Pan-American games, Asian games, Champions League, etc. Depending on the sport and the country, the effect of international competitions can be significant since the best players will not play in their domestic league program unless the calendar is adjusted accordingly. Some domestic leagues also include special events or tournaments such as the all-star weekend, the Challenge cups, or the Supercups.

Concerns around congested competitive schedules have been publicly shared across sports (Kloke, 2016; Holmes, 2018; Sport, 2020), with predominant reasons including a lack of training and recovery opportunities, and potential sleep deprivation, which can have a negative effect on the player's health (Teramoto et al., 2017; Lewis, 2018; Rossi et al., 2018) or teams' performance (Moskowitz and Wertheim, 2011; Mitchell et al., 2019; Esteves et al., 2020). Such effects could also lead to a lower product quality for consumers and broadcasters (Shelburne, 2017). Although the question of whether schedule density impacts injuries is complex, as it requires a multifaceted analysis, adjusting for many related factors such as prior injury, travel time, time zone difference, home vs. away, or acute vs. overuse injuries (Mack et al., 2018); sleep, training, and recovery opportunity are impaired due to the traveling schedule of team sports athletes (Sortino, 2015; Fullagar et al., 2016; Nutting and Price, 2017; Lastella et al., 2019). Additionally, in teams or leagues with lower budgets, or amateur sports, substantial differences in travel quality, particularly the presence of bus trips, non-charter flights, and the inevitable differences in hotel and restaurant accommodations should also be considered (Mitchell et al., 2019). Against this background, leagues have tried to modify schedules in the spirit of creating more non-game days and better traveling combinations (Holmes, 2018). Nevertheless, for especially congested periods of the season, some teams may still opt to rest players in order to provide them with extra recovery time, entailing a negative effect on the team's competitiveness and the game-product quality (Shelburne, 2017).

Appropriate training periodization and scheduling of trips and training sessions will be critical for teams to optimize training and recovery opportunity in order to maximize health and performance. This article presents a methodological framework to designing decision-support systems for scheduling in professional team sports. The proposal will follow a previously published decision support system framework (Schelling and Robertson, 2020) which considers the organization's needs, the efficiency of the processes, and the quality of the system's recommendation.

## Scheduling Problem Description

### Problem Definition

Conceptually, a team's schedule problem consists of finding a flight-and-practice schedule for the pre-season and the regular season that maximizes the opportunities to perform the periodized contents (e.g., trips, practices, games, and days off). This activity is required whilst considering known constraints (e.g., competitive schedule, roster availability, weather, special events, holidays, and emotional effect of days away). Hence, designing a schedule is a combinatorial problem, consisting of a set of instances or inputs, candidate solutions for each instance, and an overall outcome for each candidate solution (Goldreich, 2008; Mahapatra et al., 2017).

Schedule-related problems have two important features (Balas, 1999): *Constraints*, a formal description of the requirements that must be satisfied by a candidate solution to the problem; for example, a team has to be at a specific date, time and location to play the upcoming game; and an *optimization indicator*, which characterizes the quality of the recommendation. The optimization indicator represents a value whose calculation is based on the recommended solution; for example, to minimize the distance traveled in a regular season.

There are two levels of planning and scheduling depending on the time scale of decision-making. The first level “predicts” the schedule, whereas the second level “reacts” to the current local situation and is often called reactive scheduling (Aytug et al., 1994). Both levels are important; predictive scheduling is useful for macro planning (i.e., season overview), utilizing invariant information available earlier, whereas reactive scheduling should allow for enhanced decision-making thanks to better and recent information, closer to the action at hand (i.e., micro planning). Reactive scheduling is more difficult to analyze and provide meaningful automated help due to the unpredictable and recency nature of the required information to make the decision. Training session scheduling is an example of reactive scheduling, where factors such as roster availability or team performance may cause disruption in the team environment requiring a different schedule from the originally planned. Coaching and performance staff are accustomed to dealing with such disruptions. However, their decisions may be crisis-oriented or biased with little attention given to the bigger picture and impact therein (Aytug et al., 1994; Cross et al., 2019). If a computer-aided method is used for reactive scheduling it must be periodically iterated throughout the season. When new solutions require continual re-computation due to contextual changes over time the scheduling-problem is referred to as an online problem, whereas an offline problem is when information about all activities, resources, constraints and optimization indicators are known in advance, and the goal of the decision support system (DSS) is to find a single “good” solution to the problem (Wang et al., 2003).

There can be several reasons to develop a DSS for scheduling (Schelling and Robertson, 2020): the schedule simply requires application of a set of heuristic rules; the process can be automated; the current scheduling process is largely subjective or solely expertise-based; there is current disagreement among staff on how to design the schedule; new data (or criteria) allows for a re-structure of the scheduling process; team schedule has a significant impact on performance and thus warrants optimization. Additionally, when a scheduling DSS is built, the organization's knowledge about the domain becomes explicit. This enables one to study that knowledge, to critique it, to use it in training, and to preserve it over time (Fox, 1990). Last, understanding how the organization resolved scheduling-problems in the past, the available and required information-systems (hardware, software, and data workflow), the required time or deadline to solve the schedule, and the satisfaction with the implemented schedules in the past will help defining the feasibility and design of the DSS before starting its development (Schelling and Robertson, 2020).

### Constraints and Optimization Indicators

**Figure 1:**
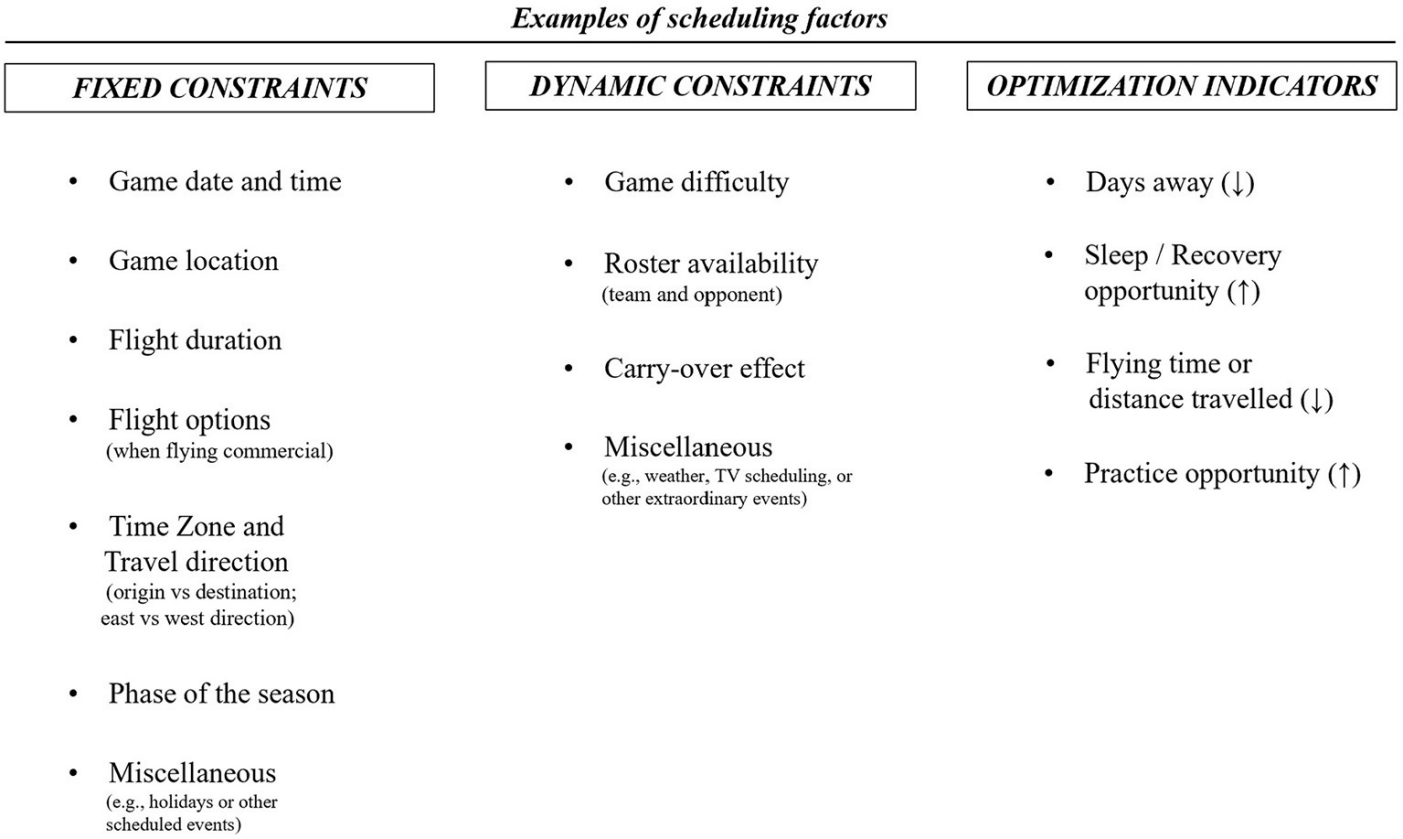
Examples of fixed and dynamic constraints, and optimization indicators relating to scheduling in professional team sport. There are potentially an infinite number of constraints and optimization indicators that could be included. Some of them are interrelated and may change over time. Different constraints and optimization indicators can be defined among various sports.

A schedule is affected by several restrictions, or constraints. These can be “fixed” (those constraints set prior to the start of the season and with none or very low variability throughout the season) or “dynamic” (those which are subject to change throughout the season) (Robertson and Joyce, 2018). Some examples of fixed constraints include the competitive calendar (game date, time, and location/topography), flight duration, flight options (when flying commercial), or time zone difference. Examples of dynamic constraints include game difficulty, standings, or roster availability (Figure 1). Some expertise-based heuristics such as preferred arrival times or accommodation preferences must be also considered as constraints when developing any DSS.

Moreover, there are schedule-problems where the goal is to optimize (maximize or minimize) an outcome variable, for instance the numbers of days away, or the distance traveled. Some examples of schedule optimization problems are spending the least possible number of days in a city with a time zone difference larger than “*x* hours,” selection of arrival time to avoid traffic in rush hours or canceling or modifying a scheduled practice session if not enough available players. In such problems the DSS will require from an optimization indicator (e.g., days away, distance traveled, recovery opportunity, practice opportunity, etc.). There are potentially an infinite number of constraints and optimization indicators that could be included, and most of them are interrelated and may change over time (Rocha, 2017) (Figure 1).

### Data Input and Sources

When developing a decision support system, data quality, including data meaning, availability, structure, integration, accessibility, and timeliness of retrieval, are critical aspects for a successful implementation (Schelling and Robertson, 2020). When direct connections (i.e., application programming interface or API) between a team's database and the league or a website's database are not available, web harvesting or scraping techniques can be explored to automate and facilitate one-time data extraction or regular feeding from online servers (Glez-Peña et al., 2014). Considering the fixed and dynamic constraint examples shown in Figure 1 below are listed some considerations regarding data input quality when developing decision support system for scheduling.

- Fixed constraints
  ○ Game location, opponent teams, dates, times, and phase of the season (pre-season, regular season, playoffs, finals, post-season) are defined by the official competitive calendar. In professional leagues the game schedule for the regular season is released several weeks before the start of the season in order to allow teams to arrange transportation and accommodation. This information is usually publicly available on each league's website (e.g., La Liga, NBA, National Football League—NFL, Major League Baseball—MLB, etc.).
  ○ International competition calendars are also made publicly available by the global governing body for each sport (FIBA, FIFA, IOC, etc.).
  ○ Geodesic distance (Karney, 2013) between cities and other travel related factors can be retrieved from public websites (e.g., www.distancecalculator.net) or automated via open source platforms.
- Dynamic constraints
  ○ Game difficulty, or win probability, considers factors such as game schedule, roster quality, home court advantage, team form, or game importance to provide a continuous (points spread) or discrete (win/lose) game outcome prediction for each team. Game difficulty can be developed internally as a sub-model within the scheduling decision support system, or retrieved from public sources (e.g., www.fivethirtyeight.com).
  ○ Daily standings and game results can be obtained from the official websites of the league, sport news websites (e.g., www.espn.com), or sport-specific sources (e.g., www.baseball-reference.com).
  ○ Daily roster availability can be retrieved from the team's athlete management system (AMS) or manually entered before the upcoming practice or game. Some sport news websites (e.g., www.espn.com) publish the injuries by team daily. Nevertheless, roster availability is often not accurate (i.e., low data quality) as there can be last-minute roster changes. Some leagues allow until 1 h before the start of the game to list a player as unavailable. Roster availability will also be affected by individual load-management needs (i.e., resting a player for a game or practice as a prophylactic strategy) (Drew and Finch, 2016), which is another example of reactive individual scheduling.
  ○ Carry-over effect, or the effect of previous events on future performance (Guedes and Ribeiro, 2011; Goossens and Spieksma, 2012) will require from integrating multiple features or even having a sub-model within the scheduling DSS.
- Data input integration refers to combining multiple sources or types of data (fixed or dynamic) to create new contextual knowledge regarding the goal at hand, thereby increasing data quality (Kenett and Shmueli, 2016). Data integration could also help optimizing the decision support system's complexity and performance, for example by reducing the data dimensionality or creating richer input features (Schelling and Robertson, 2020). Some examples are:
  ○ Schedule congestion indicators derived from game schedule (date and time) such as number of hours between games, number of games over time (e.g., number of games in 7 days, etc.), or labeling the game congestion with arbitrary categorical indicators (e.g., back-to-back, 3-in-4, or 4-in-5).
  ○ Team performance indicators based on expected performance (e.g., game difficulty or win probability) and recent performance (e.g., production in attack and defense).

**Figure 2:**
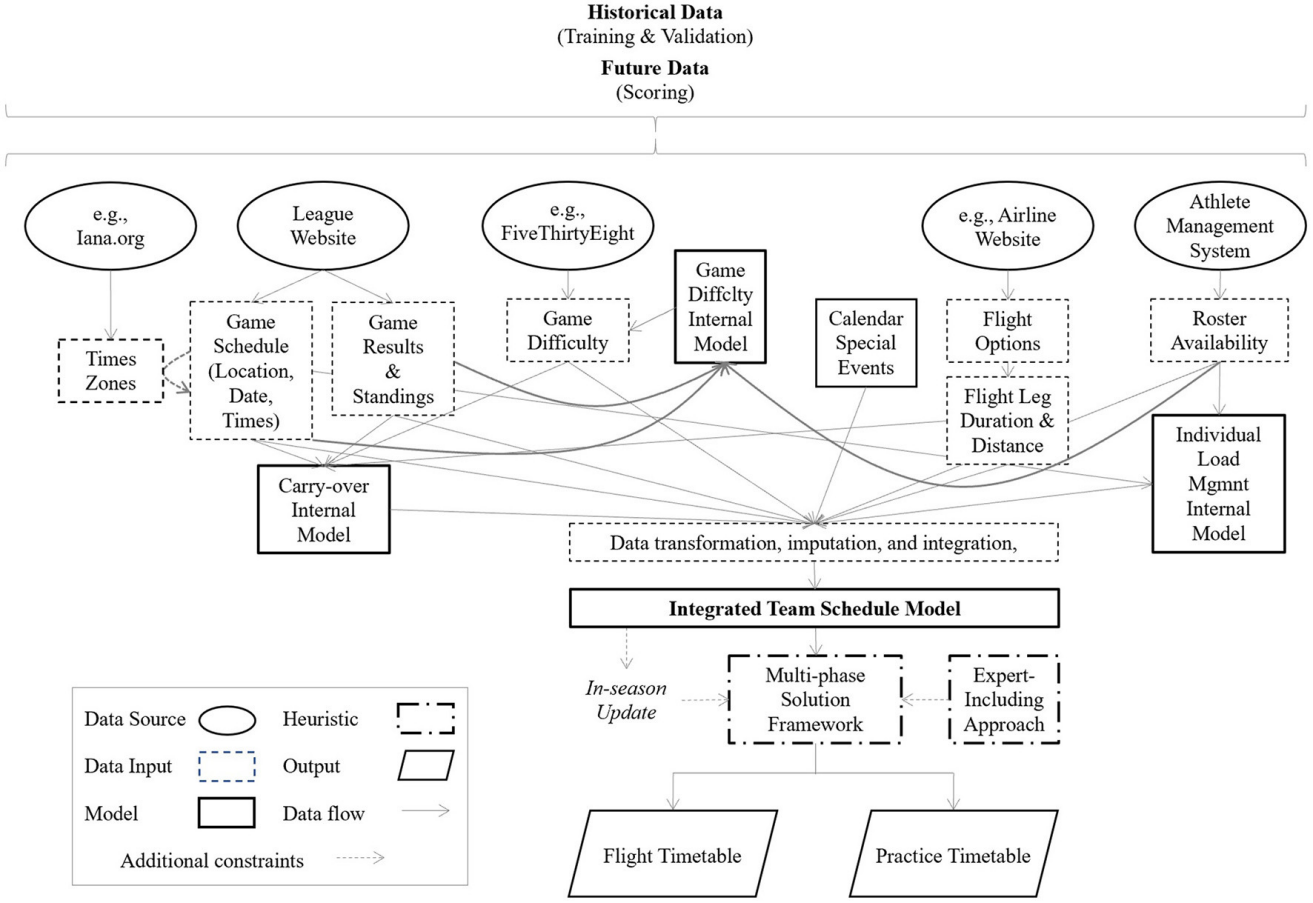
Example of the model architecture of a scheduling decision support system.

Figure 2 shows an example of model architecture including several data sources and sub-models. The example represents a multi-phase solution including different processes based on what needs to be scheduled, the available information, timescale, and the expert's knowledge:

- Phase 1: Initial competitive calendar analysis and exploration,
- Phase 2: Flight schedule recommendation,
- Phase 3: Flight schedule adjustment by expert,
- Phase 4: In-season input data update (this step can affect flight schedule also),
- Phase 5: Practice schedule recommendation,
- Phase 6: Practice schedule adjustment by expert.

### System's Decisional Guidance

The way a scheduling DSS leads users to make a decision is referred to as decisional guidance (Morana et al., 2014; Schelling and Robertson, 2020), which considers factors such as:

- What aspect of the scheduling process the system is supporting (i.e., exploration or decision),
- How explicit the output of the scheduling system is based on its delivered knowledge (i.e., description or recommendation),
- When the scheduling system provides the outcome (i.e., real-time, prospectively, or retrospectively),
- How flexible the scheduling system is (i.e., pre-defined or interactive),
- What the users' level of knowledge on scheduling and on the DSS itself is (i.e., expert or novice),
- How the output is delivered (i.e., text, tables, graphs, or image), and
- How the scheduling system is invoked (i.e., on-demand or automatically).

Appropriate decisional guidance should allow some flexibility while minimizing users' confusion and facilitating the understanding of the recommendation given by the DSS (Silver, 1991; Montazemi et al., 1996). Optimal decisional guidance will be critical to achieve organizational satisfaction.

**Figure 3:**
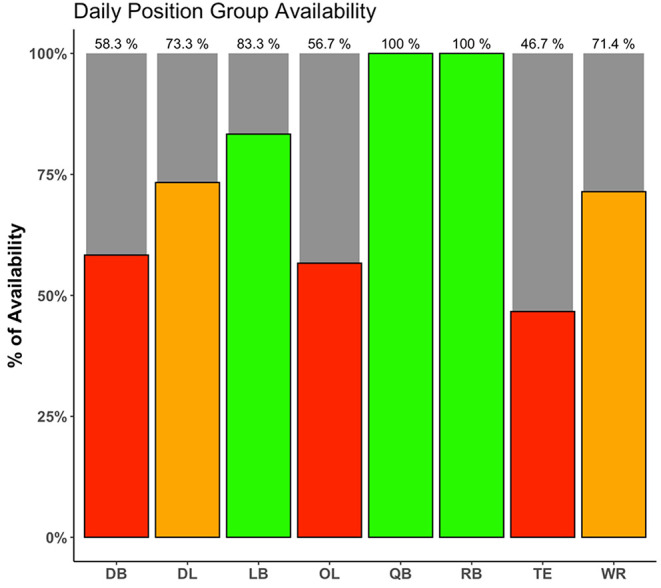
An example of a player availability report for American football, which allows coaches and staff to quickly determine which position groups have a substantial number of players unavailable for full practice, warranting a potential change in the training plan.

**Figure 4:**
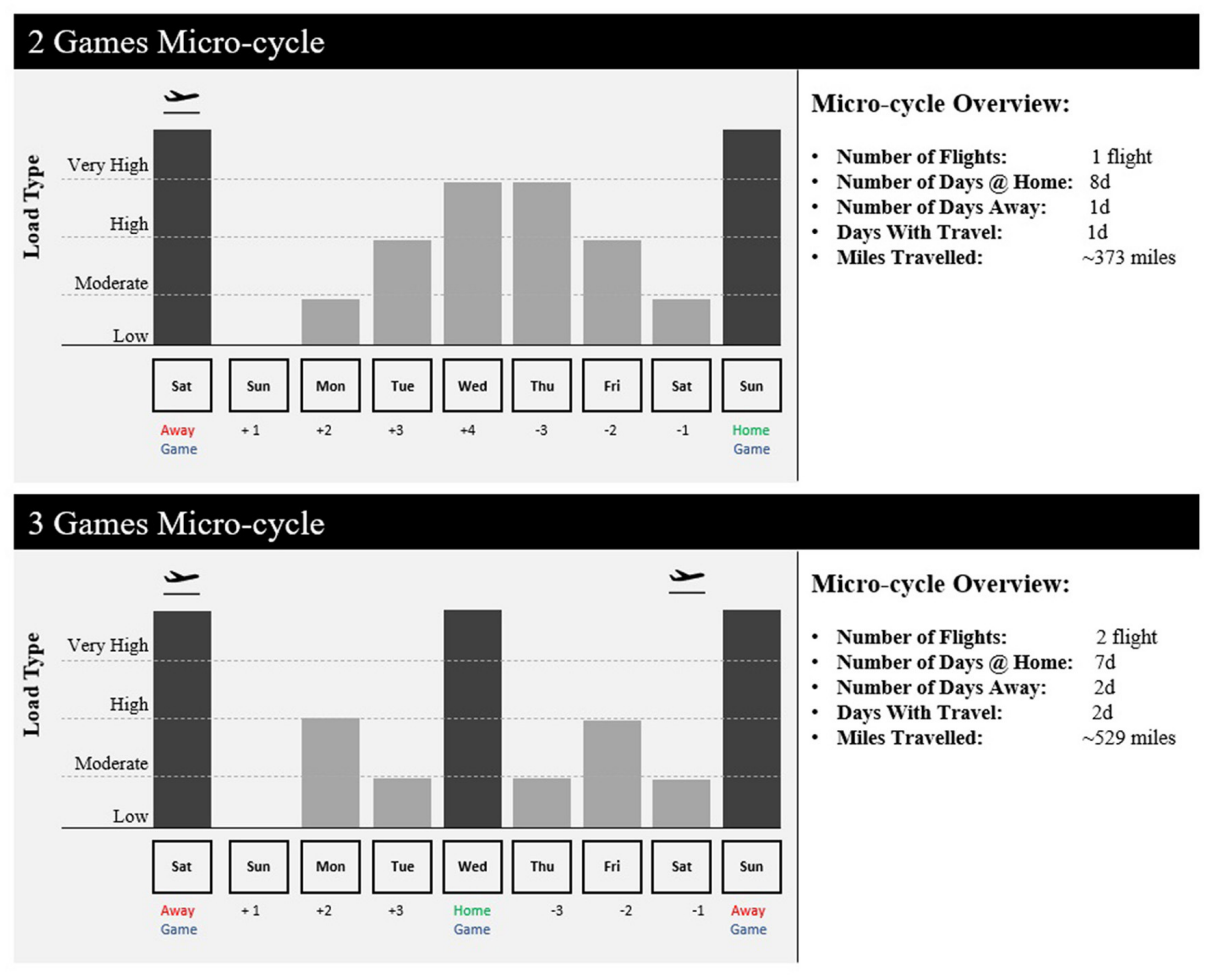
Examples of visualization of micro-cycle load distribution in soccer with different competitive calendar constraints and outputs (number of flights, number of games, number of days off, number of practice days, etc.).

**Figure 5:**
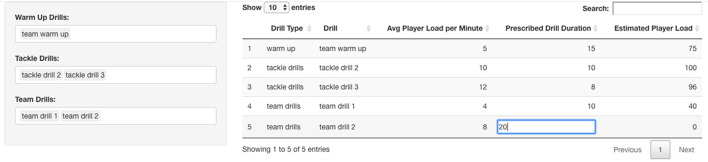
An example of session load estimator that allows the staff to build a training plan with the coach. The staff can change the drill types and manipulate the drill duration to obtain an estimation for Player Load, allowing the coaching staff to make changes to the training session for an individual athlete or position group depending on what they are able to tolerate for a given day.

Table 1 shows three examples of scheduling DSS with different decisional guidance considerations. Example 1 represents a non-interactive DSS built for a one-time schedule descriptive analysis. Example 2 shows a non-interactive DSS developed to give a recommendation on flight scheduling for the entire regular season before it starts. Example 3 represents a daily DSS, automatically invoked throughout the season, which recommends daily practice schedule for the upcoming 7 days. The daily schedule can include the roster availability (Figure 3), the official competitive calendar, a recommendation for load distribution (Figure 4), and a training session load estimator (Figure 5).

**Table 1:** Example of various decision support systems with different decisional guidance considerations.

**DSS' decisional guidance considerations**	**Example (1) non-interactive DSS for a one-time schedule descriptive analysis**	**Example (2) non-interactive DSS for one-time flight schedule before the season starts**	**Example (3) automatically invoked DSS for daily practice schedule for the upcoming 7 days**
**(1) Overall goal**	One-time research	Once-a-year automation	Daily automation
**(2) Influenced aspect of decision-making**	Overall schedule exploration	Flight schedule selection	Daily practice schedule selection
**(3) Delivered knowledge**	Information	Recommendation	Information
**(4) Output timing**	Prospective or retrospective	Prospective	Real-time
**(5) Mode**	Pre-defined	Pre-defined	Interactive
**(6) User's knowledge**	Novice	Intermediate	Expert
**(7) Communication**	Table, graphs, and map	Table, graphs, and text	Table and graphs
**(8) Invocation**	On-demand	On-demand	Automatic

*For further reading see Morana et al. (2014) and Schelling and Robertson (2020)*.

Data visualization and user interface are powerful decisional guidance tools with tremendous potential in supporting complex decision-making (Zhang and Zhu, 1998). Excellence in statistical graphics consists of complex ideas communicated with clarity, precision, and efficiency. Graphical displays should show the data; avoid distorting what the data have to say; induce the viewer to think about the substance in the project; present many numbers in a small space; make large data sets coherent; encourage the eye to compare different pieces of data; reveal the data at several levels of detail, from a broad overview to the fine structure; serve a clear purpose: decoration, description, exploration, tabulation, or recommendation; and to be closely integrated with the statistical descriptions of a data set (Tufte, 1983). Common visualization tools include charts, diagrams, drawings, graphs, ideograms, pictograms, data plots, schematics, tables, illustrations, and maps or cartograms. In scheduling-related problems there are several recurrent visualizations.

**Figure 6:**
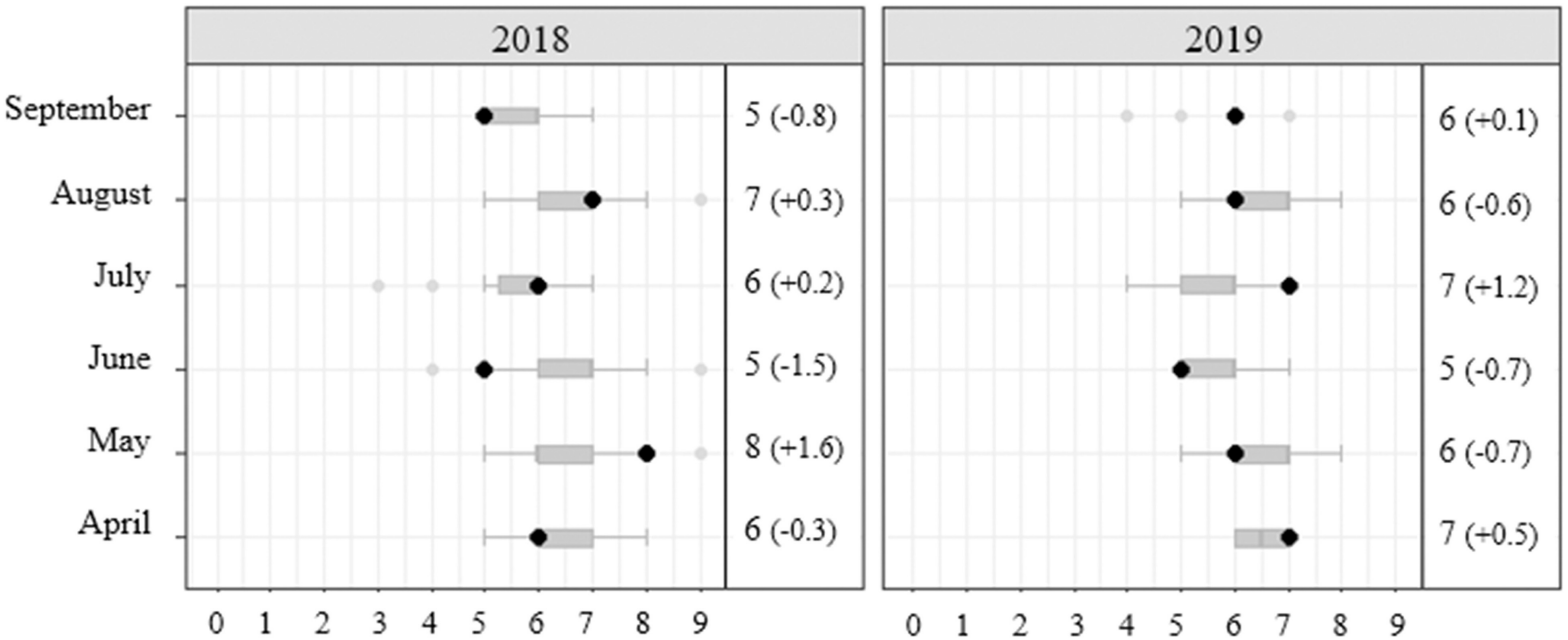
Example of visualization to explore the number of games per month (x-axis) for a Major League Baseball team (black dot) compared to the distribution of all teams in the league (gray boxplot). The differences between the team and the league's average are shown in parentheses.

**Figure 7:**
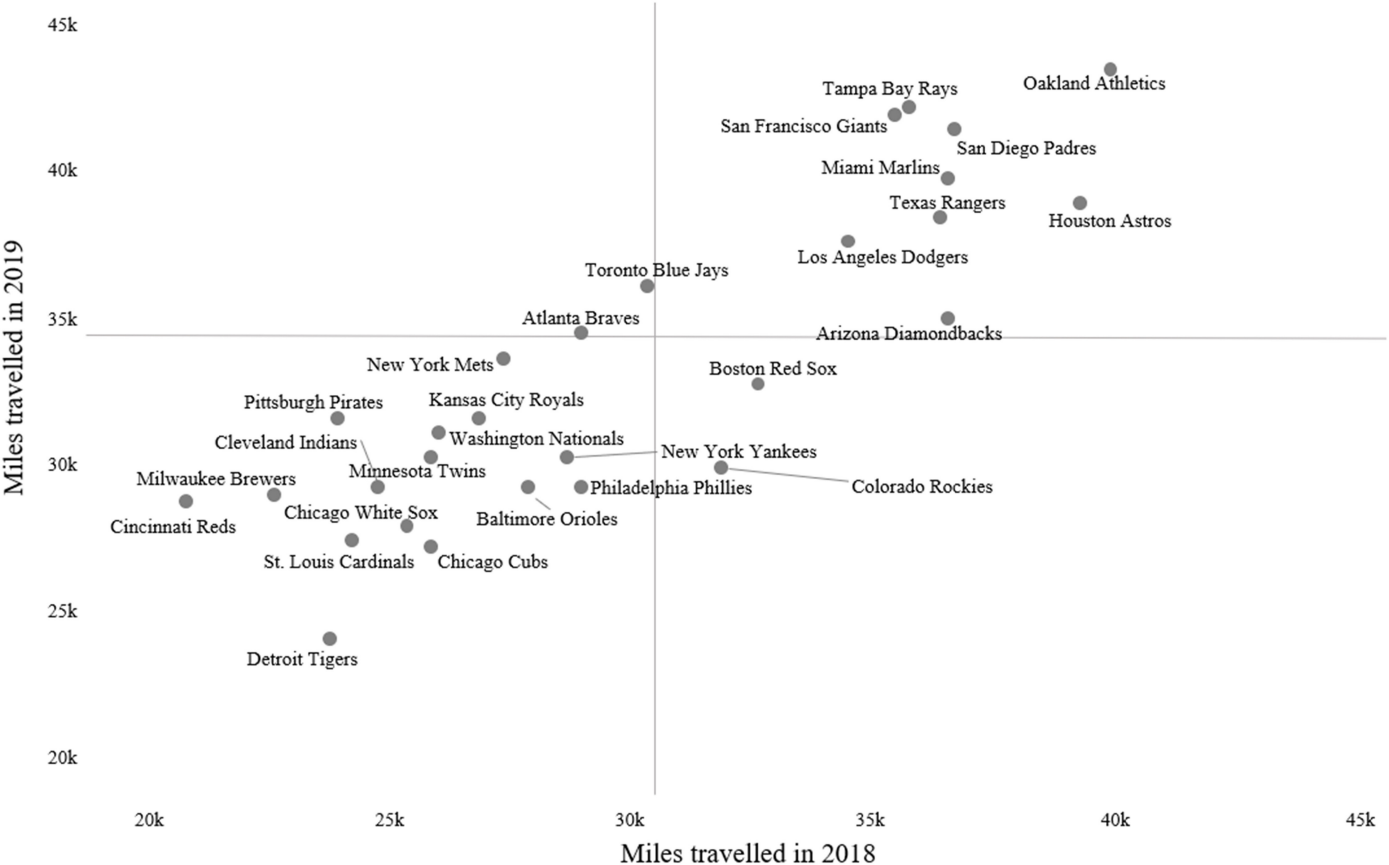
Example of a visualization representing estimated mileage traveled by Major League Baseball teams over two consecutive seasons. Reference lines represent the average mileage traveled for 2019 (horizontal reference line) and 2018 (vertical reference line).

**Figure 8:**
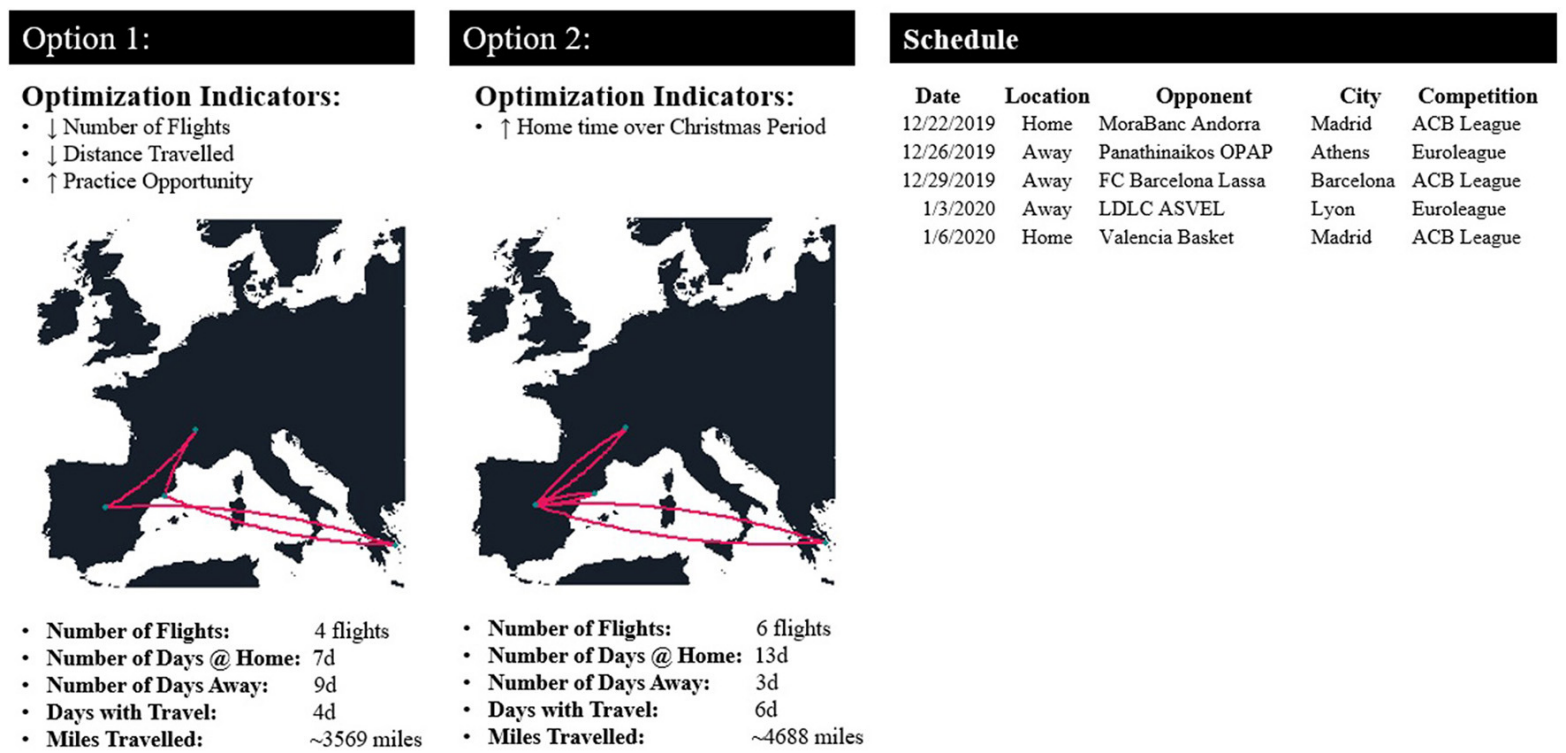
Example representing the addition of multiple “optimization indicators” and its impact on the DSS for a professional Basketball team competing in the domestic and European league over the Christmas period.

When the goal of the DSS is calendar exploration (Example 1 in Table 1), one needs to contextualize the schedule and to let the expert judge if it is good or bad compared to the rest of the teams and to previous seasons. An example would be to visualize an optimization indicator such as games played per month comparing a team against the rest of the teams, showing previous seasons as well (Figure 6). For a non-interactive DSS recommender (Example 2 in Table 1), visualizing how the optimization indicator such as distance traveled or days away compares to flight schedules from previous seasons (Figure 7) would give context for the calendar demands and the DSS' output quality. In an interactive DSS recommender (Example 3 in Table 1), visualizations could show how the modifications made by the user affect the optimization indicator, which can be multiple. For instance, changing a flight date or itinerary may increase the days away, the distance traveled, or the recovery or training opportunity (Figure 8).

In addition to calendar exploration and optimization of travel schedules, training periodization is critically important in sports with more training opportunity, with in-season micro-cycles of typically 3–7 days in duration (Akenhead et al., 2016). Coaches and support staff must not only consider the technical and tactical objectives, but also the positive (improved fitness) and negative (increased fatigue) consequences of successive training session, including pre-defined optimization indicators, and the net result on gameday (Morton et al., 1990). As recently identified by practitioners (Cross et al., 2019), there seems to be disparity between the available scientific evidence and current industry practice (i.e., human bias) in regards scheduling of training and recovery. It is here where a DSS is useful as it can provide objective contextual information and recommendations that allow practitioners to have a load distribution overview for the upcoming micro-cycle (Figure 4) as well as to prescribe training sessions (Figure 5) considering individual needs within a team structure (i.e., reactive scheduling). This process will be mainly constrained by the competitive calendar (e.g., number of games, location, day of the week, and time) (Akenhead et al., 2016) and the players' availability (Hagglund et al., 2013). Information from the Athlete Management System (AMS) can be retrieved to determine which players are injured and will be unavailable for training in the upcoming week, which players need additional recovery time following the last game, and which players are able to participate in full. Codifying these details allows the staff to identify training loads and position groups that may be challenged to have enough players available to train on a given day. Such information can be reflected in a dashboard or web application, allowing coaches to make any necessary changes to the weekly training plan should certain positional groups be at risk due to a limited number of players being able to participate in full (Figure 3). Finally, once the micro-cycle structure has been designed and the available players identified, a customized session load estimator can be used to help adjust the practice and make it more appropriate considering the micro-cycle load distribution and the available players. Figure 5 shows an example of a session load estimator that allows the support staff to build the training session with the coach and manipulate the drill duration to automatically get an estimation of player load [e.g., the sum of instantaneous rate of change of acceleration, or jerk, divided by a scaling factor (Nicolella et al., 2018)] for a given session. Tools such as this aid the decision making of the staff as drills can be removed or added from the session and training duration for a specific drill can be altered to gain an understanding of the potential training demands on a position group or individual for the upcoming session.

## Scheduling Models

Traditional approaches to solving scheduling problems use either simulation models, analytical models, heuristic approaches or a combination of these methods (Aytug et al., 1994; Balas, 1999; Mahapatra et al., 2017). Simulation models are primarily used to assess schedules and are most useful for schedule exploration (e.g., initial competitive calendar analysis and tentative flight dates) (Aytug et al., 1994). Analytical models include mathematical programming models, stochastic models, and control theory approaches focusing on optimization processes. A disadvantage of these models is that the problem needs to be explicitly formulated, which is difficult for schedulers who do not have the mathematical knowledge and background (Zhou et al., 2013). Additionally, since even the most simplified scheduling problems are complex, realistically sized problems cannot be optimally solved, and real-life applications of analytical approaches are scarce (Aytug et al., 1994). Consequently, a wide body of heuristic approaches have been investigated to find near-optimal solutions in cases where finding the optimal solution is impractical (Zhou et al., 2013; Mahapatra et al., 2017). Some research has shown that human interactions with automated heuristics methods often offer improved performance (Aytug et al., 1994). Computer-based systems are better than humans at finding complex and subtle patterns in massive data sets, but humans are very effective connecting different sources of information in creative and unpredictable ways (Akata et al., 2020). DSS offers a mean to combine various types of knowledge in a manner that can be used for scheduling problems (Schelling and Robertson, 2020).

**Figure 9:**
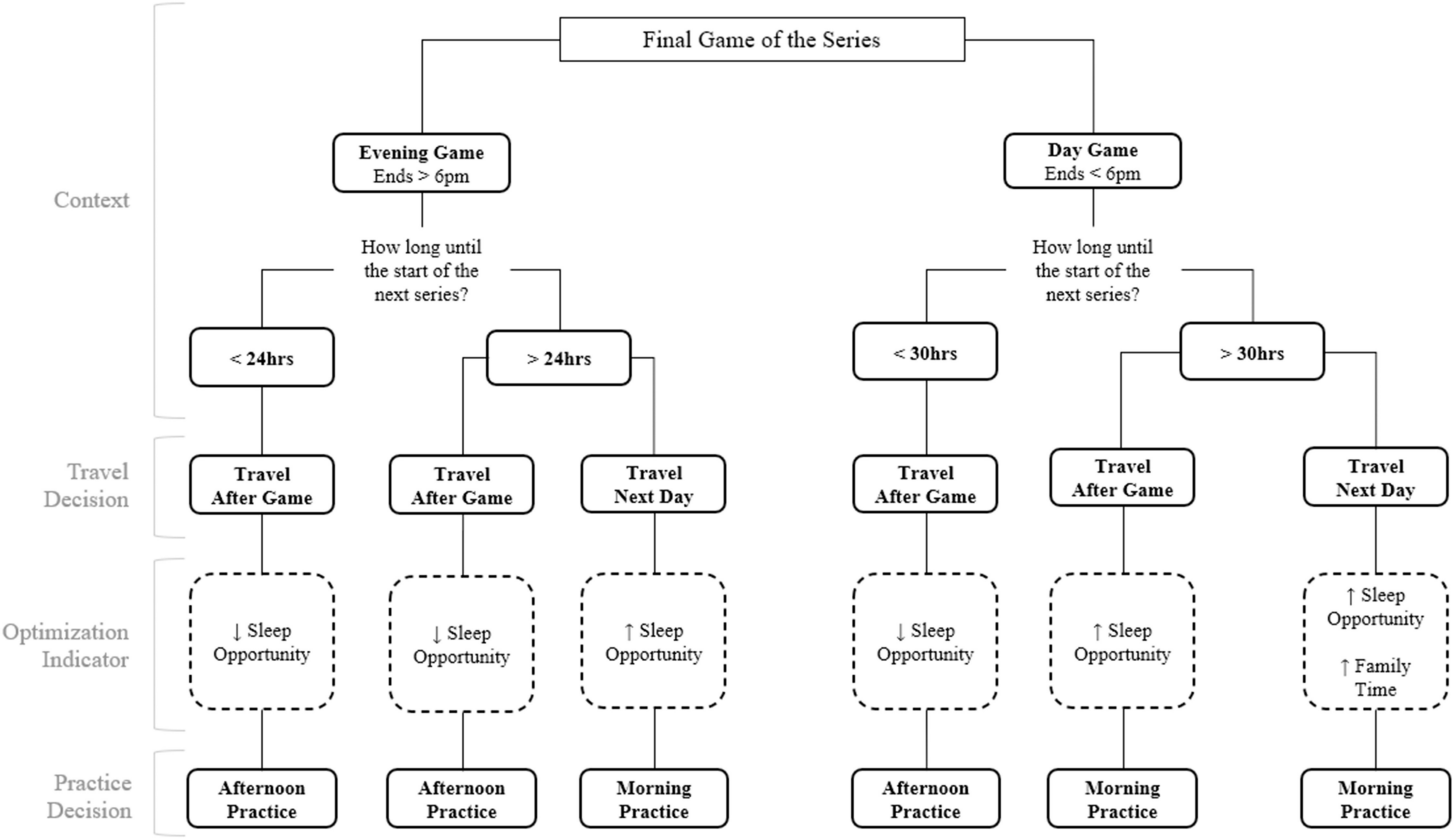
Example of expert system (ES) applied to Major League Baseball.

Expert systems (ES) represent a special case of knowledge-based scheduling DSS (Aytug et al., 1994). ES are developed by first acquiring the knowledge from a human expert and then codifying this knowledge into a series of algorithmic rules (Figure 9). Scheduling ES can recommend decisions on actual or simulated cases and do so in a way that captures the idiosyncratic nature of a specific organization. Nevertheless, many researchers (Aytug et al., 1994) believe that expert system approaches are not ideal for scheduling because most real-life environments present complex relationships that are often difficult to model with simple association rules. Two additional issues are that most environments are so dynamic that knowledge becomes obsolete too fast (Fox and Smith, 1985), and that the input of a small set of experts might focus too strongly on specific individual experience, hindering the generalization capabilities of the model. Consequently, more advanced computer-based approaches such as random search, blind search or heuristic search have been implemented for scheduling problems. Constraint-based heuristic search are methods that use knowledge about the restrictions, or constraints, of the scheduling problem to guide and limit the search of a near-optimal solution within a search space that is too large to explore entirely (Trick et al., 2012). Nevertheless, a limitation of many computer-based methods in scheduling is their inability to adapt to changing demands without human-intensive intervention. This observation has led to including learning components in scheduling DSS. Machine learning methods focus on learning from experience to provide predictions on yet-unobserved data, without requiring human intervention in the learning process, and, in many cases, being able to adapt when new data is available.

For the scheduling problem in sports, both supervised (e.g., regressions, decision trees, support vector machine, *K*-nearest, random forest) and unsupervised (e.g., clustering, PCA) machine learning algorithms could provide a mechanism for creating better features to be used as input for the scheduling DSS (see Song et al., 2019 for more on the interaction between machine learning and optimization processes). Some examples of richer features include the difficulty level estimation of a game, the estimation of a team's carry-over effect throughout the season or discretizing continuous variables that are difficult to model within a DSS such as player load (see the three sub-models in Figure 2).

Besides the computational complexities and requirements, the desired decisional guidance discussed in the previous section, requires several design considerations when choosing the analytical processes and techniques embedded in the system. The system's acceptance and its outcome interpretability will be related to the selected model architecture (Ribeiro et al., 2016). Selection of one family of algorithm over another may also change, when possible, the way in which the problem is framed for the end user (Schelling and Robertson, 2020). The scheduling DSS should aim for the most efficient and effective analytical process to solve a task while it meets the interpretability and the operational functions expected by the end-user. Developers need to design a DSS that can provide an understanding of any discrepancy between the DSS recommendation and the expert's opinion (identification of expert bias) (Kayande et al., 2009). Many standard machine learning algorithms such as logistic regression, decision trees, decision-rules learning, or *K*-nearest neighbors are examples of more interpretable algorithms, whereas random forest, gradient boosting, support vector machine, neural networks and deep learning fall into the less- or non-interpretable machine learning approaches (i.e., black-box algorithms) (Luo et al., 2019). When a black-box model produces significantly better recommendations than a more interpretable model, the scheduling DSS developer may consider integrating feedback within the system (Kayande et al., 2009), with tools such as partial dependence (PD) plots, individual conditional expectation (ICE), local interpretable model-agnostic explanation (LIME), or kernel Shapley values (SHAP) to help partially understand the scheduling recommendation and to ensure trust and transparency in the decision process of the model (Messalas et al., 2019). On the other hand, if there are no specific design needs of relying on the mentioned black-box methods as the main model for the DSS their capacity of exploiting non-linear relationships could still be used to derive richer features, such as the ones mentioned above. Another data-based approach that could provide a good balance between interpretability and prediction accuracy is the use of probabilistic graphical models (e.g., Bayesian networks), which would allow practitioners to obtain a clearer idea of the relationship between the different variables within the DSS and inspect the impact that one decision might have in the rest of the variables. A potential issue of probabilistic outputs and visualizations is that humans generally have more difficulty understanding these than frequency-based data with familiar units (Tversky and Kahneman, 1983).

## Scheduling Decision Support System Evaluation

When it comes to evaluate how the scheduling DSS is performing, three overarching aspects need to be reviewed: context satisfaction, process efficiency, and output quality. The first consideration refers to how satisfied the organization is with the system (e.g., is the DSS covering the organization's needs? is it technically and economically feasible?). The second aspect refers to the efficiency of the process (e.g., is the DSS user-friendly? Is the recommendation given by the DSS what the end-user expected? Is the complexity of the model adequate? Is the interpretation of the recommendation clear for the user?). The third and last criterion relates to the quality of the recommendation (e.g., is the recommended schedule been followed on its entirety by the organization? if not, how many instances have been modified? if there was an optimization indicator, did the DSS' recommendation improve historical decisions? is the DSS capable of learning based on the expert modifications?). Based on these three considerations a comprehensive DSS evaluation tool has been previously published (Schelling and Robertson, 2020), which includes feasibility, decisional guidance, data quality, system complexity, and system error as the assessment components. Nevertheless, assessing a scheduling system's error might seem cumbersome, but as discussed on the section on decisional guidance, assessing the system's output quality will require a subjective and an objective perspective. For instance, Figure 8 shows two scheduling options based on different optimization indicators (physiological and psychological). The expert will find more suitable one option than the other for the team's context. Visualizing the degree of agreement between the scheduling DSS recommendation and the expert's decision can help evaluating the overall DSS recommendation quality, in addition to the analysis of the optimization indicators when the DSS recommendation are changed. Future research should include analyzing the efficacy of scheduling DSS on enhancing decision-making processes and key performance indicators (KPIs).

## Conclusion

A scheduling decision support system can enhance a schedule better than a human-judgment-only approach primarily by automating certain or all processes, by objectively weighing constraints in the schedule (i.e., optimization), and allowing systematic historical comparisons, particularly if personnel changes occur. Scheduling DSS can include predictive and exploratory solutions for macroplanning (e.g., competitive calendar analysis and tentative travel schedule), and reactive solutions for microplanning (e.g., weekly session prescription and travel updates). These solutions must consider several contextual constraints (fixed and dynamic) and provide the nearest-optimal solution, since an optimal solution might not be feasible due to contextual requirements or computational complexity. Constraints and optimization indicators, as well as the advantages of the DSS adoption may differ between organizations. An integrative understanding of current scheduling practices and the organization's needs prior to the development of the DSS is warranted. Traditional approaches to solving scheduling problems use either simulation models, analytical or mathematical models, heuristic approaches, or a combination of these methods. Machine learning algorithms (supervised and unsupervised) could provide a mechanism for creating better features to be used as input (e.g., game difficulty, carry-over effect, and discretization of continuous variables) or for reducing data dimensionality (i.e., variable selection). For a better acceptance and a successful implementation, the scheduling DSS recommendation process should be as understandable as possible. Visualization techniques might be required to improve the system's interpretability. Once implemented, the system's recommendations (output) and the users' feedback (interaction) can be closely and systematically monitored for eventual improvements.

## Data Availability Statement

The original contributions presented in the study are included in the article/supplementary material, further inquiries can be directed to the corresponding author/s.

## Author Contributions

XS: conception, design, drafting, critical revision, visuals, and final approval of the papers' version to be published. SR: critical revision and final approval of the papers' version to be published. JF, PW, and JF: critical revision, feedback, and visuals. All authors contributed to the article and approved the submitted version.

## Conflict of Interest

The authors declare that the research was conducted in the absence of any commercial or financial relationships that could be construed as a potential conflict of interest.
